# A simplified bedside model using age, NRS-2002, and ADL to predict postoperative delirium in elderly hip fracture patients: a retrospective cohort study

**DOI:** 10.3389/fpsyt.2026.1963389

**Published:** 2026-10-19

**Authors:** Qinyuan Chen, Shanping Chen, Lijuan Guan, Yaoxuan Wu, Zuowei Pei

**Affiliations:** Geriatric Diseases Institute of Chengdu, Department of Geriatrics, Chengdu Fifth People's Hospital (The Second Clinical Medical College, Affiliated Fifth People's Hospital of Chengdu University of Traditional Chinese Medicine), Chengdu, China

**Keywords:** elderly, hip fracture, nomogram, NRS-2002, postoperative delirium, prediction model

## Abstract

**Background:**

Postoperative delirium (POD) is the most common neuropsychiatric complication after hip fracture surgery in elderly patients. Existing prediction models rely on cognitive assessments or intraoperative parameters that are difficult to obtain in emergency settings. This study aimed to develop and internally validate a simplified model using age, Nutritional Risk Screening 2002 (NRS-2002), and Activity of Daily Living (ADL) dependency to predict POD.

**Methods:**

This single-center retrospective cohort study included patients aged >=60 years who underwent hip fracture surgery at Chengdu Fifth People’s Hospital between January 2019 and August 2023. Patients with pathological fractures or pre-existing delirium were excluded. Multiple imputation by chained equations (MICE, m=20) was used for missing data, with pooled estimates combined using Rubin’s rules. To prevent data leakage, all imputation models were fitted strictly within the training folds of cross-validation, and all internal validation metrics were computed on out-of-fold (OOF) predictions. Discrimination was assessed by the area under the receiver operating characteristic curve (AUC) with 5-fold stratified cross-validation repeated 20 times; calibration was assessed by the calibration slope, calibration-in-the-large, the Hosmer-Lemeshow test, and the Brier score. Complete-case analysis and delta-adjusted MNAR sensitivity analyses were performed.

**Results:**

A total of 387 patients were included, with 43 (11.1%) developing POD. All three variables were independent predictors: age (OR = 1.096, 95% CI: 1.039-1.157, P<0.001), NRS-2002 (OR = 2.075, 95% CI: 1.370-3.142, P<0.001), and ADL (OR = 1.593, 95% CI: 1.094-2.319, P = 0.015). The cross-validated AUC was 0.815 (95% CI across repeats: 0.810-0.819; pooled OOF AUC 0.812), with a calibration slope of 1.068 (95% CI: 0.885-1.252), Brier score of 0.0848, and Hosmer-Lemeshow P = 0.086. At the optimal cutoff (0.115), sensitivity was 88.4% (38/43) and the negative predictive value reached 97.9%, with an observed POD rate of 2.1% among patients below the cutoff; the positive predictive value was 24.8%. The complete-case analysis (N = 200, 21 events) was underpowered, prone to selection bias, and is reported purely as a directional sensitivity check.

**Conclusion:**

This simplified three-variable model demonstrates moderate discrimination and acceptable calibration. With an NPV of 97.9% but a modest PPV of 24.8%, it is best suited as a bedside rule-out screening tool rather than for identifying high-risk patients. The NRS-2002 estimate attenuated under MNAR sensitivity analysis and is interpreted with caution. Multicenter prospective external validation, incorporating systematic cognitive screening, is needed before clinical application.

## Introduction

Hip fractures are among the most burdensome traumatic injuries in aging societies, with an estimated 1.5 million new cases annually worldwide, projected to reach 4.5 million by 2050 (1, 2). Postoperative delirium (POD) is the most common neuropsychiatric complication after hip fracture surgery, with incidence ranging from 11% to 62% (3, 4). POD is not merely a transient disturbance of consciousness; it systematically prolongs hospitalization, increases mortality, and accelerates cognitive decline (5, 6). Despite its clinical importance, POD prediction in elderly hip fracture patients remains inadequate. Existing models typically embed preoperative cognitive assessments (e.g., MMSE), laboratory parameters, or intraoperative variables that are impractical in time-constrained emergency settings (7–10). In this study, we sought to develop a simplified rule-out screening model using only information immediately available at the bedside upon emergency admission. Age was chosen as a non-modifiable biological baseline; NRS-2002 provides a composite nutritional risk assessment (incorporating BMI, recent weight loss, dietary intake, and disease stress) that nursing staff can complete within minutes (11); and the ADL dependency score reflects pre-fracture functional status and frailty, routinely recorded during nursing admission assessment. All three variables can be obtained without additional testing, cognitive evaluation, or laboratory results, making the model practical for time-pressed emergency settings. We prioritized high negative predictive value to safely identify low-risk patients who may not require intensive delirium prevention measures.

## Materials and methods

### Study design and population

This single-center retrospective cohort study included patients aged >=60 years who underwent hip fracture surgery at Chengdu Fifth People’s Hospital between January 2019 and August 2023. Patients with pathological fractures or pre-existing delirium at admission were excluded. Of 670 consecutive admissions screened, 283 without any postoperative CAM documentation were excluded, leaving 387 patients in the final cohort (**Figure 1**); nine of these admissions occurred before 2020 (six in 2019 and three with implausibly recorded admission dates in 2002) and lacked NRS-2002 and ADL documentation entirely. The study was approved by the Ethics Committee of Chengdu Fifth People’s Hospital (Lunshen 2023-049 (Ke)-01, approved December 11, 2023), and informed consent was waived due to the retrospective nature. This study was reported in accordance with the TRIPOD (Transparent Reporting of a multivariable prediction model for Individual Prognosis or Diagnosis) statement (12).

**Figure 1:**
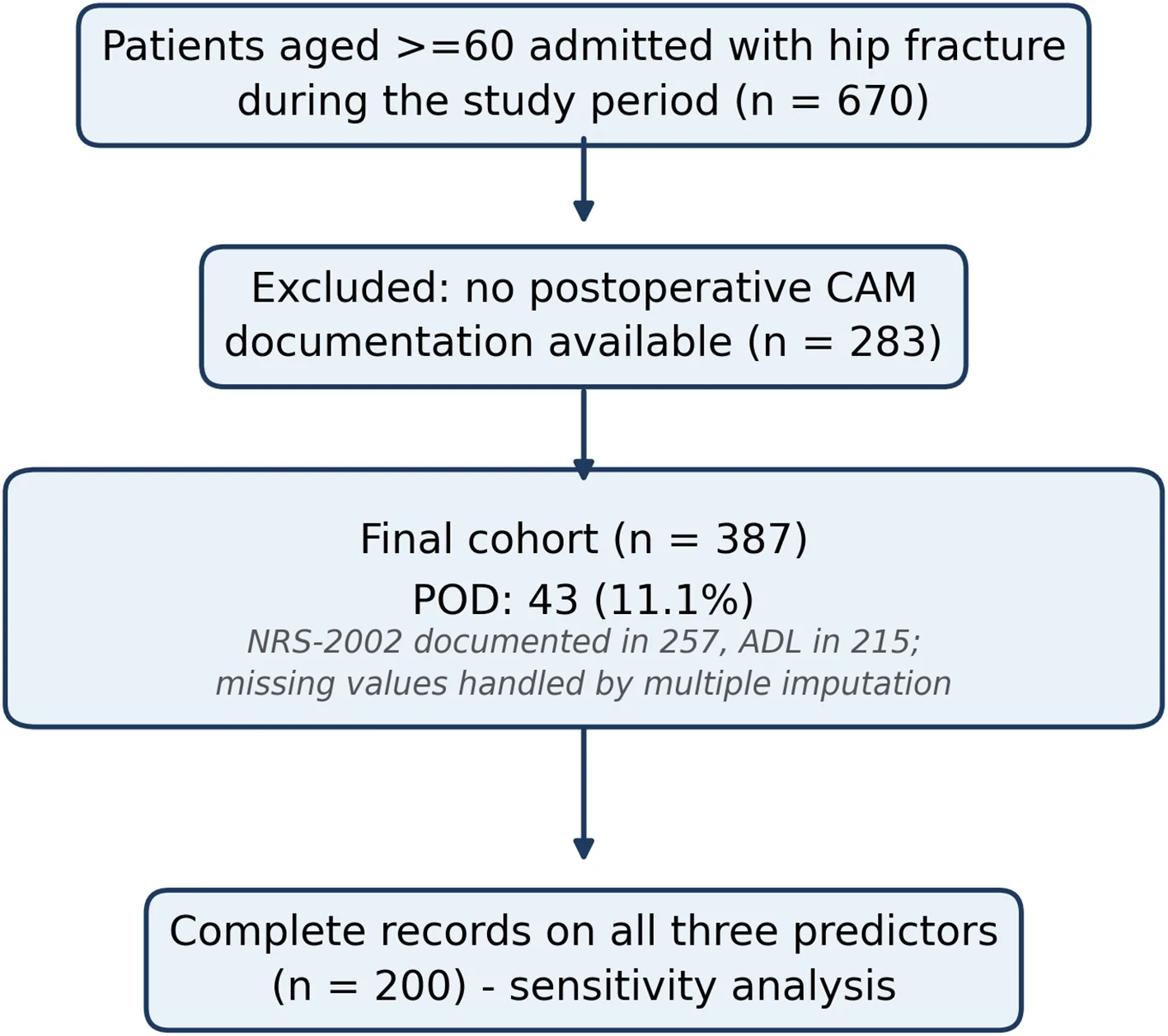
Flow of patients through the study. Of 670 consecutive admissions aged >=60 years undergoing hip fracture surgery, 283 without postoperative CAM documentation were excluded; the final cohort comprised 387 patients, of whom 200 had complete records on all three predictors (sensitivity analysis).

### Outcome and predictors

POD was defined according to the Confusion Assessment Method (CAM) and ascertained from medical records. Postoperative CAM evaluations were performed by physicians who had completed unified standardized training and held certification in comprehensive geriatric assessment; assessments were triggered by clinical indication and supplemented by routine documentation of mental status in nursing and medical records. The surveillance window extended from surgery to discharge (median postoperative length of stay, 12 days; IQR 9-16).

Three candidate predictors were fixed *a priori* based on clinical accessibility at the time of emergency admission: age; NRS-2002 score (range 0-7; higher indicates greater nutritional risk) (11); and ADL dependency score (range 0-4; 0=fully independent, 4=fully dependent). All three are captured in the routine admission process for essentially every geriatric admission in our ward, require no laboratory work, and can be completed within minutes at the bedside. The selection was made on accessibility grounds before examining outcome associations; it was not based on a formal systematic review and was not pre-registered.

### Missing data

NRS-2002 was missing in 130/387 (33.6%) and ADL in 172/387 (44.4%); age was complete. Both scores are components of the institutional preoperative comprehensive geriatric assessment (CGA) program, which was progressively rolled out during the study period and achieved full documentation coverage from 2023 onward. The patterns and correlates of missingness are quantified in **Supplementary Tables S1**, **S2**, and the year-by-year rollout is reported in the Results.

### Statistical analysis

Multiple imputation by chained equations (MICE) was implemented via scikit-learn IterativeImputer (version 1.3) with BayesianRidge conditional models (13). Imputation models included the three predictors and the outcome variable to preserve predictor-outcome associations; imputed values were truncated to the valid score range and rounded to integers. We acknowledge that chained-equation imputation of ordinal scores via linear conditional models with rounding is an approximation; this is addressed by the sensitivity analyses below. The number of imputations was set to m=20, exceeding the recommended minimum of the largest percentage of missing data (44.4%) (14). Pooled estimates were combined using Rubin’s rules. The distributions of observed and imputed values were compared across imputed datasets and are reported in the **Supplementary Materials**.

Because IterativeImputer does not implement features of reference MICE software such as predictive mean matching, we additionally re-estimated the model using a full chained-equation multiple imputation procedure with bootstrap perturbation of imputation-model coefficients and residual resampling (outcome included, m=20) to confirm that the conclusions did not hinge on the implementation choice.

Internal validation used 5-fold stratified cross-validation, repeated 20 times with a fixed random seed. To eliminate the risk of data leakage, imputation was performed strictly within each training fold: in every fold, the imputation models (with the outcome included) were fitted on the training portion only and then applied to the held-out fold; the 20 within-fold imputations were averaged into out-of-fold (OOF) predictions. Every internal validation metric reported below - including the cross-validated AUC (mean and 95% CI across the 20 repeats, t-distribution), calibration metrics, the optimal cutoff, and decision curve analysis - was computed on OOF predictions, so no additional optimism correction was required. The apparent AUC and its 95% CI (1,000 stratified bootstrap resamples) are reported separately as in-sample reference values.

Calibration was assessed by the calibration slope (logistic regression of the outcome on the logit of predicted risk, no intercept), calibration-in-the-large (intercept of the same model with intercept), the expected-to-observed (E:O) ratio, the Brier score, and the Hosmer-Lemeshow test on deciles of predicted probability; a calibration curve with bootstrap 95% confidence bands was constructed. The optimal cutoff was determined by maximizing the Youden index on OOF predictions. Decision curve analysis (DCA) was likewise based on OOF predictions.

Sensitivity analyses comprised: (1) a complete-case analysis (N = 200, 21 events, EPV = 7.0), interpreted purely as a directional sensitivity check rather than as a test of the missing-at-random assumption, given its underpowered event count and its susceptibility to selection bias (differences between complete and incomplete patients are quantified in **Supplementary Table S2**); and (2) delta-adjusted MNAR analyses, in which imputed values of NRS-2002 and ADL were shifted upward by 1 and 2 points (toward worse nutritional and functional status) before model re-estimation (**Supplementary Table S3**).

The sample size was assessed against the events-per-variable (EPV) criterion, with a minimum threshold of 10 (15). All analyses used Python 3.12 (pandas 2.2, statsmodels 0.14, scikit-learn 1.3), with two-sided P<0.05 considered significant.

## Results

### Study population

A total of 387 patients were included (**Figure 1**), with 43 (11.1%) developing POD and 344 (88.9%) not. The mean age was 80.8 +/- 8.1 years (range 60-101), and 260 (67.2%) were female. Among these, 200 had complete data on all three variables (21 POD events) for sensitivity analysis.

### Baseline characteristics

Patients who developed POD were significantly older (85.6 +/- 5.7 vs. 80.2 +/- 8.2 years, SMD = 0.76, P<0.001), had higher NRS-2002 scores (4.1 +/- 0.8 vs. 3.3 +/- 1.0, SMD = 0.93, P<0.001), and greater ADL dependency (2.6 +/- 1.3 vs. 1.5 +/- 1.3, SMD = 0.83, P<0.001) compared to those without POD (**Table 1**).

**Table 1:** Baseline characteristics of POD and non-POD groups (MICE full sample, N = 387).

Variable	POD (n=43)	Non-POD (n=344)	P value
Age, years (mean+/-SD)	85.6+/-5.7	80.2+/-8.2	<0.001
Female, n (%)	27 (62.8)	233 (67.7)	0.541
BMI, kg/m2 (mean+/-SD)	21.3+/-3.4	22.6+/-3.5	0.041
NRS-2002 score (mean+/-SD)	4.1+/-0.8	3.3+/-1.0	<0.001
ADL score (mean+/-SD)	2.6+/-1.3	1.5+/-1.3	<0.001
Hypertension, n (%)	31 (72.1)	239 (69.5)	0.753
Diabetes, n (%)	12 (27.9)	92 (26.7)	0.876
Coronary heart disease, n (%)	15 (34.9)	98 (28.5)	0.486
Chronic heart failure, n (%)	11 (26.2)	42 (12.2)	0.029
Cerebrovascular disease, n (%)	10 (23.3)	52 (15.1)	0.231
Chronic kidney disease, n (%)	8 (18.6)	41 (11.9)	0.347
COPD, n (%)	7 (16.3)	41 (11.9)	0.487
Parkinson, n (%)	3 (7.0)	12 (3.5)	0.463
Osteoporosis, n (%)	29 (69.0)	193 (57.3)	0.184
Cognitive impairment, n (%)	9 (21.4)	27 (8.0)	0.011
Comorbidity count, median (IQR)	2 (2-2)	2 (2-2)	0.145

SMD, Standardized Mean Difference; IQR, Interquartile Range. NRS-2002 range 0-7 (higher=greater risk). ADL range 0-4 (0=fully independent, 4=fully dependent).

### Missing data patterns

Missingness of NRS-2002 (33.6%) and ADL (44.4%) tracked the staged rollout of the preoperative CGA program: ADL documentation was absent for 100% of the 2020 admissions (n=120), 37.3% in 2021 (n=110), 2.1% in 2022 (n=96), and 0% in 2023 (n=51), and the nine pre-2020 admissions lacked both scores entirely (**Supplementary Table S1**). Patients with complete records were comparable to those with incomplete records in age (80.9 +/- 7.9 vs. 80.7 +/- 8.3 years, P = 0.761), female sex (64.5% vs. 70.1%, P = 0.292), and POD incidence (10.5% vs. 11.8%, P = 0.815) (**Supplementary Table S2**). Among patients with partially documented scores, recorded NRS-2002 values were higher (3.7 vs. 3.3, P<0.001) and ADL grades worse (2.3 vs. 1.6, P = 0.040) in patients lacking the other score; both patterns are examined by the sensitivity analyses below.

### Model performance

Univariable logistic regression (**Table 2**) showed associations of age (OR = 1.099, 95% CI: 1.049-1.151), NRS-2002 (OR = 2.243, 95% CI: 1.506-3.342), and ADL (OR = 1.750, 95% CI: 1.262-2.427) with POD (all P<0.001). In the multivariable MICE model (**Table 3**), all three variables remained independent predictors: age (OR = 1.096, 95% CI: 1.039-1.157, P<0.001), NRS-2002 (OR = 2.075, 95% CI: 1.370-3.142, P<0.001), and ADL (OR = 1.593, 95% CI: 1.094-2.319, P = 0.015). The pooled logistic regression equation (MICE m=20, Rubin’s rules) was: logit(P) = -13.37 + 0.0921 x Age + 0.7299 x NRS-2002 + 0.4653 x ADL, where P is the predicted probability of POD. The overall model likelihood ratio test was significant (median-P rule across imputations, P<0.001), and the Nagelkerke R-squared was 0.241.

**Table 2:** Univariable logistic regression for POD (MICE full sample, N = 387).

Variable	OR	95% CI	P value	Univariable AUC
Age (per year)	1.099	1.049-1.151	<0.001	0.699
NRS-2002 (per point)	2.243	1.506-3.342	<0.001	0.773
ADL (per grade)	1.750	1.262-2.427	<0.001	0.760

**Table 3:** Multivariable logistic regression for POD (MICE m=20, Rubin’s rules, N = 387).

Variable	Adjusted OR (95% CI)	Wald chi-square	P value
Age (per year)	1.096 (1.039-1.157)	12.6	<0.001
NRS-2002 (per point)	2.075 (1.370-3.142)	15.5	<0.001
ADL (per grade)	1.593 (1.094-2.319)	9.3	0.015

Model likelihood ratio test (median-P rule): P<0.001; Nagelkerke R-squared=0.241.

The apparent AUC was 0.845 (95% CI: 0.781-0.896; **Figure 2**), and the cross-validated AUC was 0.815 +/- 0.009 (95% CI across the 20 repeats: 0.810-0.819; pooled OOF AUC 0.812), an optimism gap of 0.030. Calibration of OOF predictions was acceptable (**Figure 3**): calibration slope 1.068 (95% CI: 0.885-1.252), calibration-in-the-large 0.455 (95% CI: -0.302-0.867), E:O ratio 0.983 (mean predicted 11.3% vs. observed 11.1%), Brier score 0.0848, and Hosmer-Lemeshow chi-square=13.85 (df=8, P = 0.086).

**Figure 2:**
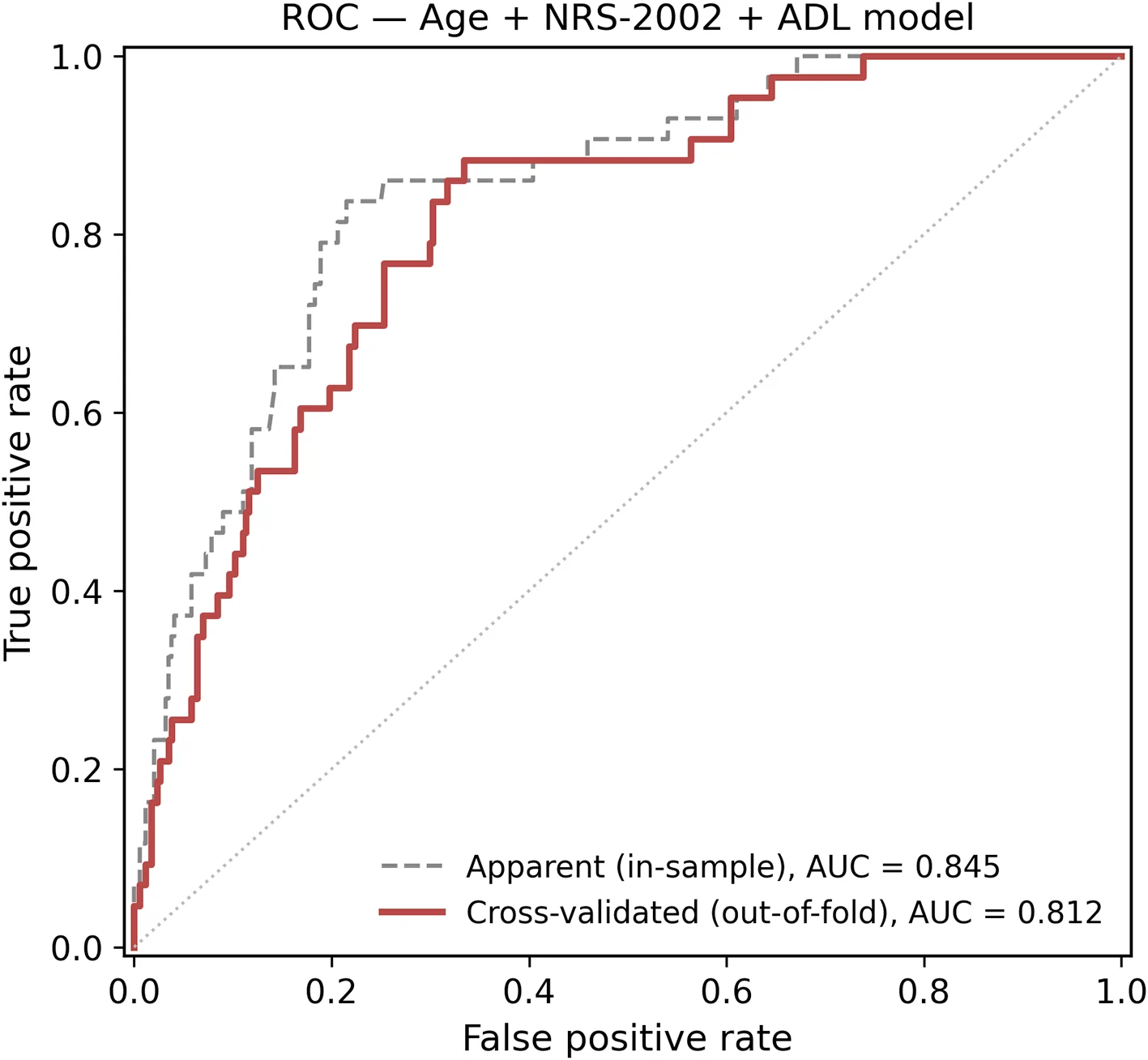
Receiver operating characteristic (ROC) curves of the prediction model. Dashed grey line: apparent (in-sample) performance, AUC = 0.845 (95% CI: 0.781-0.896, bootstrap n=1000). Solid red line: cross-validated out-of-fold performance pooled over 20 imputations and 20 repeats of 5-fold cross-validation, AUC = 0.812. The optimal cutoff (0.115; sensitivity=88.4%, specificity=66.6%) was determined on out-of-fold predictions.

**Figure 3:**
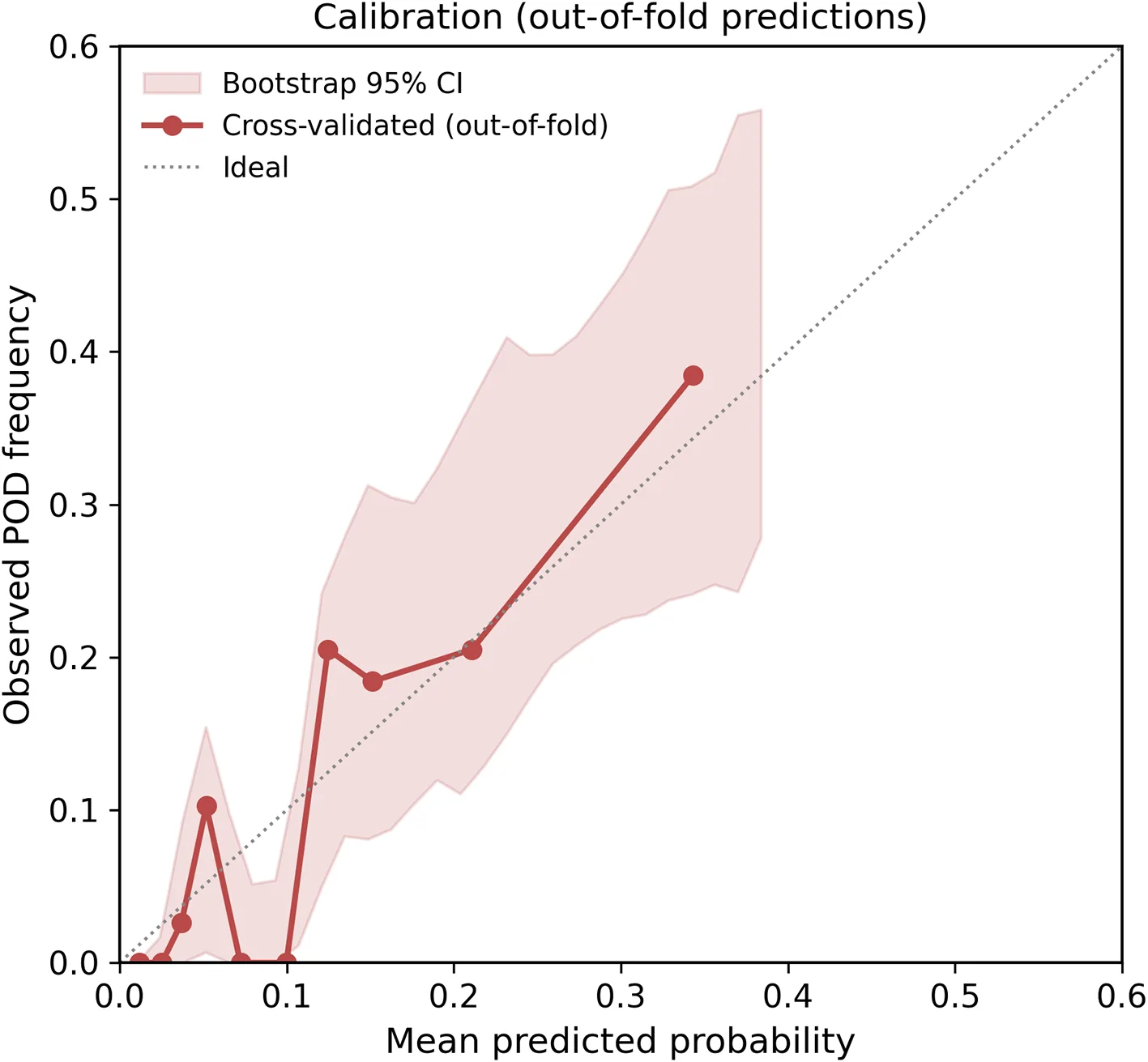
Calibration of out-of-fold predictions. The calibration slope was 1.068 (95% CI: 0.885-1.252) and calibration-in-the-large was 0.455 (95% CI: -0.302-0.867); the Brier score was 0.0848 and the Hosmer-Lemeshow test P = 0.086. Shaded band: bootstrap 95% confidence interval.

At the optimal cutoff (0.115, Youden J = 0.550; **Figure 4**), sensitivity was 88.4% (38/43), specificity 66.6% (229/344), PPV 24.8%, and NPV 97.9%. Among the 234 patients classified as low-risk below the cutoff, 5 developed POD, corresponding to an observed POD rate of 2.1%. DCA based on OOF predictions showed positive net benefit across threshold probabilities of 5%-49% (**Figure 5**); OOF-predicted probabilities in this cohort ranged from 0.2% to 62% (median 8.7%), so thresholds beyond this range have no practical interpretation for a screening rule in this population, and below 5% the model did not separate from a treat-none strategy at this prevalence.

**Figure 4:**
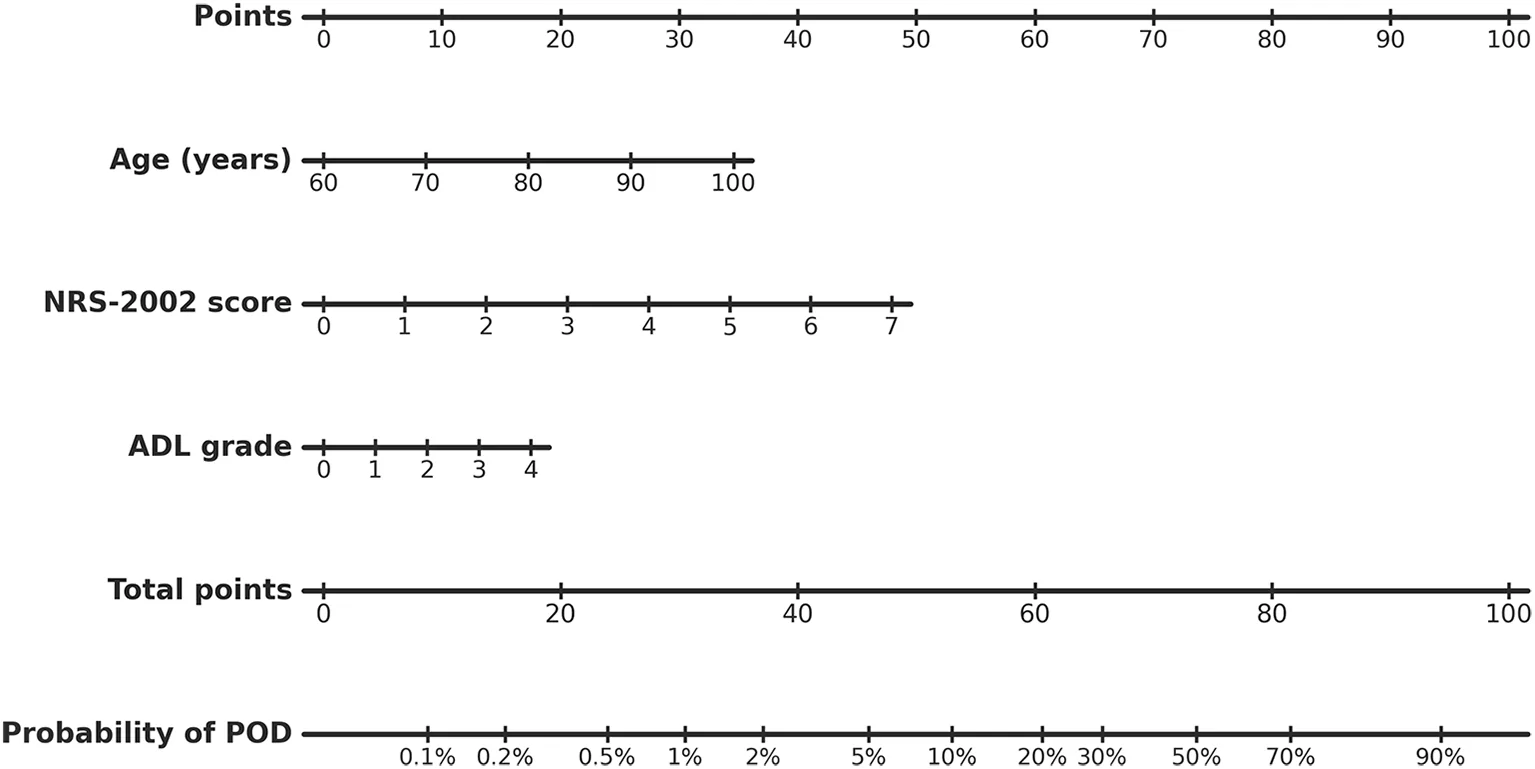
Nomogram for predicting postoperative delirium, reconstructed from the final pooled coefficients (logit(P) = -13.37 + 0.0921 x Age + 0.7299 x NRS-2002 + 0.4653 x ADL). Points for each predictor are read against the top scale (0-100). The probability axis is an exact logistic transform of the linear predictor; the achievable range of total points (0-100) corresponds to probabilities from below 0.1% up to approximately 94%, and the axis is labelled accordingly. To use: locate the patient’s age, NRS-2002, and ADL scores on their respective axes, read the corresponding points, sum them, and locate the total on the probability axis.

**Figure 5:**
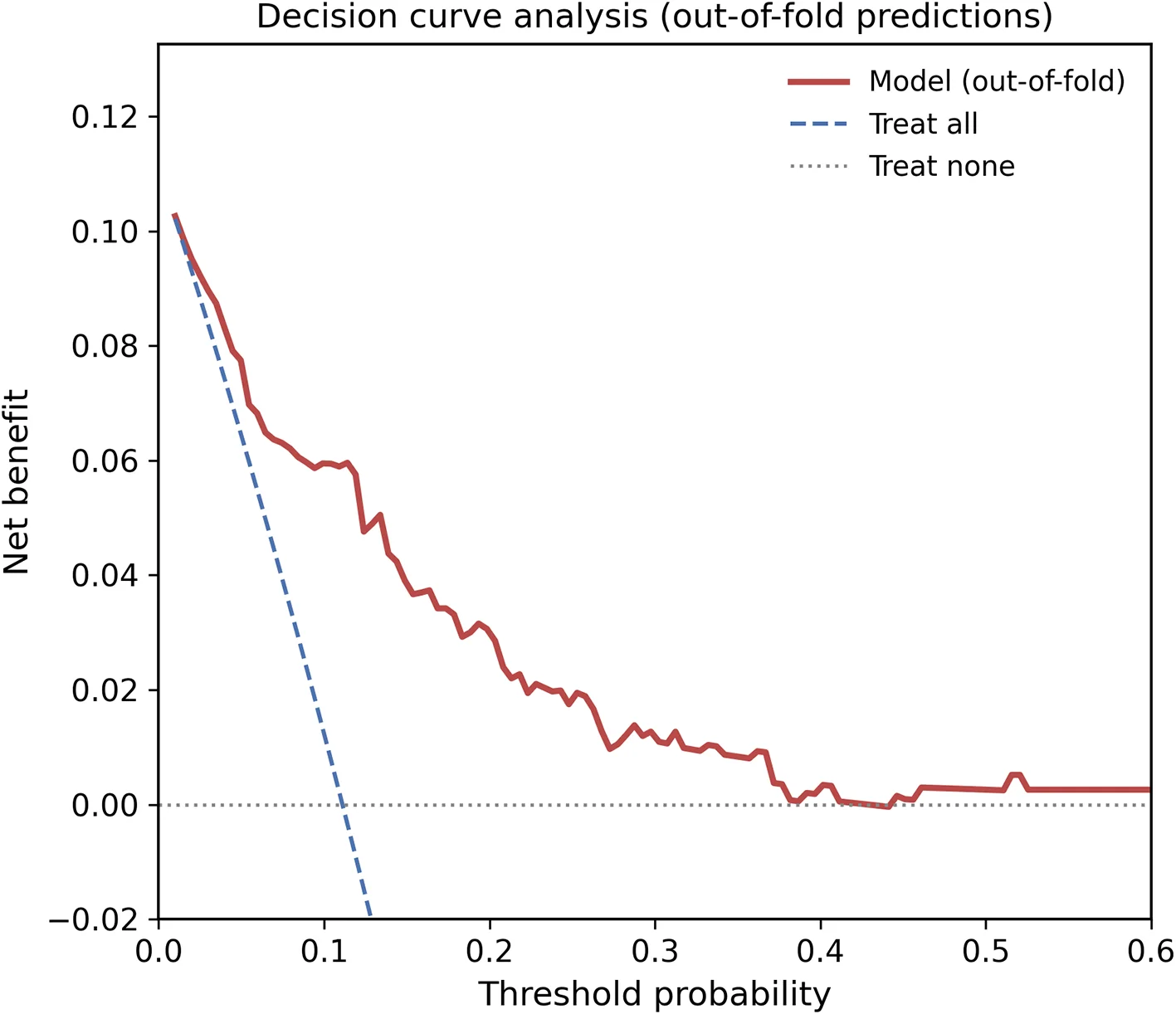
Decision curve analysis based on out-of-fold predictions. The blue line represents the prediction model, the red dashed line represents “treat all,” and the black dotted line represents “treat none.” The model provided positive net benefit across threshold probabilities of 5%-49%; out-of-fold predicted probabilities ranged from 0.2% to 62% (median 8.7%), so thresholds beyond this range have no practical interpretation in this population.

### Sensitivity analyses

The full chained-equation MI with bootstrap perturbation yielded pooled estimates consistent with the primary analysis: age OR = 1.100 (95% CI: 1.040-1.164), NRS-2002 OR = 2.855 (95% CI: 1.682-4.845), and ADL OR = 1.861 (95% CI: 1.271-2.724), all P<0.001; conclusions were unchanged under both implementations.

Under delta-adjusted MNAR analysis (**Table 4** note), the age and ADL estimates were robust to progressively worse-shifted imputed values (age OR 1.096->1.095->1.095; ADL OR 1.593->1.548->1.478), whereas the NRS-2002 estimate attenuated (OR 2.075->1.552->1.204) while remaining directionally positive.

**Table 4:** Performance of the primary MICE model and the complete-case sensitivity analysis.

Metric	MICE primary (N = 387)	Complete case (N = 200)
Apparent AUC (95% CI)	0.845 (0.781-0.896)	0.881 (0.810-0.950)
Cross-validated AUC (95% CI across 20 repeats)	0.815 (0.810-0.819)	0.851 (0.846-0.856)
Pooled out-of-fold AUC	0.812	0.856
Calibration slope (95% CI)	1.068 (0.885-1.252)	0.928 (0.678-1.179)
Calibration-in-the-large (95% CI)	0.455 (-0.302-0.867)	-0.247 (-0.982-0.489)
Hosmer-Lemeshow chi-square (df=8)	13.85, P = 0.086	16.44, P = 0.037
Brier score (out-of-fold)	0.0848	0.0714
Optimal cutoff (out-of-fold, Youden)	0.115	0.123
Sensitivity/Specificity at cutoff	88.4%/66.6%	81.0%/81.6%
PPV/NPV at cutoff	24.8%/97.9%	34.0%/97.3%

MNAR delta-adjustment (primary model, OR at delta=0/+1/+2): Age 1.096/1.095/1.095; NRS-2002 2.075/1.552/1.204; ADL 1.593/1.548/1.478.

The complete-case analysis (N = 200, 21 POD events, EPV = 7.0) yielded apparent AUC 0.881 (95% CI: 0.810-0.950), cross-validated AUC 0.851 +/- 0.010 (95% CI across repeats: 0.846-0.856; pooled OOF AUC 0.856), calibration slope 0.928 (95% CI: 0.678-1.179), calibration-in-the-large -0.247 (95% CI: -0.982-0.489), Brier score 0.0714, and Hosmer-Lemeshow chi-square=16.44 (df=8, P = 0.037) (**Table 4**). Consistent with its pre-specified interpretation, this analysis was underpowered and prone to selection bias; its nominally higher discrimination is attributable to sampling instability with only 21 events, and its nominally suboptimal calibration further underscores that no performance claim rests on it.

## Discussion

This study developed and internally validated a simplified three-variable model for POD prediction in elderly hip fracture patients. Using MICE with 20 imputations and Rubin’s rules, with all imputation strictly confined to training folds to eliminate leakage, the model achieved a cross-validated AUC of 0.815 with acceptable calibration. The NPV of 97.9% at the optimal cutoff - an observed POD rate of 2.1% among patients below it - suggests the model is best suited as a rule-out screening tool: patients classified as low-risk have a very low observed probability of developing POD, allowing preventive resources to be directed to higher-risk patients.

The NRS-2002 score was the strongest predictor (OR = 2.075), consistent with meta-analytic evidence that malnutrition is an independent risk factor for POD (16). NRS-2002 captures a composite of recent weight loss, dietary intake decline, and disease stress, which single-dimensional indices (e.g., albumin, BMI) may miss (17, 18). This estimate, however, warrants explicit caution. Under delta-adjusted MNAR sensitivity analysis, shifting imputed values toward worse nutritional status attenuated the NRS-2002 estimate from 2.075 to 1.204 while age and ADL remained essentially unchanged. Because imputed NRS-2002 values for unassessed patients rely on the missing-at-random assumption, the true strength of the NRS-2002 association may lie toward the lower end of this range; the direction of the association was preserved in all scenarios. The ADL dependency score (OR = 1.593) likely proxies for frailty and cognitive reserve, both established POD risk factors (19). The Nagelkerke R-squared of 0.241 indicates that these three variables explain a modest proportion of POD variance, consistent with the multifactorial nature of delirium. This supports the model’s positioning as a screening tool rather than a comprehensive risk assessment.

Mazzola et al. (7) reported an apparent AUC of 0.84 using a 7-variable model (including MMSE and MNA-SF). Our model achieved a cross-validated AUC of 0.815 with only 3 variables, with the difference partly attributable to validation method (apparent vs. cross-validated estimates) and to the different predictor sets. We emphasize that the three predictors were fixed *a priori* on grounds of accessibility at the moment the prediction is needed - age, NRS-2002, and ADL are captured for essentially every emergency geriatric admission in our ward, whereas cognitive screening, laboratory panels, and detailed medication reconciliation are not. The choice was therefore a design decision rather than a data-driven one; univariable associations are reported descriptively in **Table 2** and did not govern predictor selection. In univariable comparison, chronic heart failure (P = 0.029) and documented pre-existing cognitive impairment (P = 0.011) were associated with POD in our cohort, but neither was systematically or uniformly assessed in the retrospective source: a documented cognitive-impairment flag reflects impairment noticed by the treating clinician rather than a systematic screen, so incorporating it as a predictor would misclassify precisely the un-diagnosed mild cognitive impairment and dementia cases that matter most, and several other candidate domains (pre-fracture function, psychoactive and anticholinergic medication, fracture type, time to surgery, anemia, infections, anesthesia type) are inconsistently documented. With 43 events, adding these domains would trade a complete three-variable model (EPV = 14.3) for a partial and unstable one. The model should accordingly be read as a preliminary bedside screening model, not a comprehensive etiological risk model; multi-variable prospective cohorts are needed to test whether the omitted domains add performance.

Several limitations merit emphasis. First, this was a single-center retrospective study with internal validation only; the estimates require external validation before clinical use, and the nine pre-2020 admissions with implausible or early dates were retained under multiple imputation, which we report transparently. Second, the complete-case sensitivity analysis (N = 200, 21 events, EPV = 7.0) was underpowered, prone to selection bias (**Supplementary Table S2**), and is interpretable purely as a directional sensitivity check; no performance claim rests on it. Third, although missing-data comparisons showed no differences in age, sex, or POD between complete and incomplete patients, the MAR assumption for imputed NRS-2002 and ADL cannot be verified empirically. Reassuringly, the missingness pattern tracked the institutional rollout of the CGA program (ADL missingness 100% in 2020 to 0% in 2023) rather than patient characteristics, making patient-level selection less likely to be the dominant mechanism; nevertheless, delta-adjusted MNAR analyses showed that the NRS-2002 estimate is sensitive to departures from MAR, and the Discussion interprets it accordingly. Fourth, POD ascertainment relied on retrospective CAM documentation: assessments were performed by physicians with unified standardized training and certification in comprehensive geriatric assessment, triggered by clinical indication and supplemented by routine mental-status documentation, over a surveillance window from surgery to discharge. This approach risks missing hypoactive delirium episodes, which would bias estimates toward the null, so the reported associations are likely conservative; differential misclassification cannot, however, be excluded, and conclusions are worded accordingly. Fifth, baseline cognition was not systematically screened in this cohort - there was no uniform MoCA or MMSE administration at admission - so the model could not include cognitive status despite its univariate association (P = 0.011); prospective cohorts incorporating systematic cognitive screening are a prerequisite for external validation.

Future work should pursue multicenter prospective validation with protocolized daily screening, systematic cognitive assessment, and standardized predictor collection; given the strong and now carefully qualified NRS-2002 signal, trials of targeted nutritional intervention in high-risk patients are a natural next step.

## Conclusion

In conclusion, this simplified three-variable model demonstrated moderate discrimination (cross-validated AUC 0.815) and acceptable calibration, with an NPV of 97.9% and an observed POD rate of 2.1% below the cutoff. It is best suited as a bedside rule-out screening tool: a low-risk classification can support triage of preventive resources, while a high-risk classification should prompt heightened vigilance rather than be taken as diagnostic. External validation is needed before clinical application.

## Data Availability

The original contributions presented in the study are included in the article/**Supplementary Material**. Further inquiries can be directed to the corresponding author.
