# Supplementary material for: A simplified bedside model using age, NRS-2002, and ADL to predict postoperative delirium in elderly hip fracture patients: a retrospective cohort study
Source: Front Psychiatry. 2026 Oct 19;17:1963389. doi: 10.3389/fpsyt.2026.1963389 (PMC13725447; doi:10.3389/fpsyt.2026.1963389)
Supplement: Supplementary file 1 [file SupplementaryFile1.docx]

**Supplementary Tables**

Supplementary Table S1. Cohort composition and missingness by admission year. NRS-2002 and ADL are components of the institutional preoperative comprehensive geriatric assessment (CGA) program, which was rolled out in stages during the study period; patients without the assessment record are missing both scores. Nine admissions before 2020 (six recorded in 2019, three with implausibly early admission dates) were retained; all lacked NRS-2002 and ADL documentation. One admission with no recorded admission date could not be assigned to a year (NRS-2002 missing, ADL documented).

| **Admission year** | **n** | **POD, n (%)** | **NRS-2002 missing, n (%)** | **ADL missing, n (%)** |
| --- | --- | --- | --- | --- |
| 2002 | 3 | 0 (0.0%) | 3 (100.0%) | 3 (100.0%) |
| 2019 | 6 | 2 (33.3%) | 6 (100.0%) | 6 (100.0%) |
| 2020 | 120 | 15 (12.5%) | 102 (85.0%) | 120 (100.0%) |
| 2021 | 110 | 19 (17.3%) | 4 (3.6%) | 41 (37.3%) |
| 2022 | 96 | 7 (7.3%) | 14 (14.6%) | 2 (2.1%) |
| 2023 | 51 | 0 (0.0%) | 0 (0.0%) | 0 (0.0%) |
| Date not recorded | 1 | 0 (0.0%) | 1 (100.0%) | 0 (0.0%) |
| Total | 387 | 43 (11.1%) | 130 (33.6%) | 172 (44.4%) |

Supplementary Table S2. Comparison of patients with complete vs incomplete predictor data (N=387). Group differences in age, sex, and POD were tested with t-test/chi-square as appropriate.

| **Variable** | **Complete (n=200)** | **Incomplete (n=187)** | **P value** |
| --- | --- | --- | --- |
| Age, years (mean±SD) | 80.9±7.9 | 80.7±8.3 | 0.761 |
| Female, n (%) | 129 (64.5) | 131 (70.1) | 0.292 |
| POD, n (%) | 21 (10.5) | 22 (11.8) | 0.815 |
| NRS-2002 among those recorded (mean±SD) | 3.3±0.9 | 3.7±1.2 | <0.001 |
| ADL among those recorded (mean±SD) | 1.6±1.2 | 2.3±1.3 | 0.040 |

Supplementary Table S3. MNAR delta-adjustment sensitivity analysis (primary MICE model). Imputed values were shifted upward by delta points toward worse status before re-estimation. The NRS-2002 effect attenuates with increasing delta while remaining directionally positive; age and ADL are robust.

| **Predictor** | **OR (delta=0)** | **OR (delta=+1)** | **OR (delta=+2)** |
| --- | --- | --- | --- |
| Age | 1.096 | 1.095 | 1.095 |
| NRS-2002 | 2.075 | 1.552 | 1.204 |
| ADL | 1.593 | 1.548 | 1.478 |
